# Revitalize Primary Health Care for Confronting Current Public Health Challenges

**DOI:** 10.4103/0970-0218.39233

**Published:** 2008-01

**Authors:** Rajesh Kumar

**Affiliations:** PGIMER School of Public Health, Chandigarh - 160 012, India. E-mail: dr.rajeshkumar@gmail.com

Thirty years ago, in 1978, the World Health Organization (WHO) espoused the Primary Health Care strategy to achieve the goal of ‘Health for All’. Since then, considerable progress has been made in population health around the world, particularly in developing countries. However, required investments have not been made and the full potential of primary healthcare has not been realized. In the last three decades, unprecedented technological and political changes that swept the world have ushered in an era of ‘globalization’. The influence of the ‘market’ is growing and the long-cherished public health values of social solidarity are under serious threat. Macroeconomic policies are squeezing public spending on social services in several countries. The weakening of industrial regulations is not only threatening the health of the working population but is also leading to environmental crises in many communities. Trade liberalization and the extended reach of mass media advertising are encouraging the consumption of unhealthy products on a large scale. Rapidly expanding global travel is providing newer opportunities to microbes to affect communities far away from their habitat. Traditional governance structures are increasingly losing relevance and the comprehensive primary healthcare strategy stands abandoned.

Keeping society healthy is the prime task of Community Physicians. That is why rather than only managing patients in cozy clinics, they are always busy in planning, organizing or evaluating Health Promotion or Disease Control Programs. And the principal task of those who are engaged in academic community medicine is to produce a Health Workforce which is not only capable of protecting but also has competency in promoting people's health. Community physicians have always been interested in knowing why some communities or various groups within the same community are healthier than others. The prime focus of this investigation has been to find out the causes of ill health. The physical and/or social conditions in which people live and work, described as the determinants of health, have been established as the underlying causes of health inequalities around the world. However, rather than arguing for fundamental change in the social policies for promoting health, community medicine has largely adopted a technocentric approach to disease prevention and control during the last century. This approach has been successful to some extent in prolonging life but the goal of ‘health for all’ by the turn of the century remained largely unfulfilled.

In these extraordinary times, revitalization of primary healthcare approach for promoting health, protecting the environment, controlling disease and making healthcare accessible to all has become a big challenge. To confront emerging challenges, primary healthcare concepts and approaches need to be re-emphasized. The Health Field Concept,(1) Alma Ata Declaration,(2) the Ecological Perspective(3) and Health Promotion Strategy(4) have enriched our discipline. Shelter, food, water, income, a stable ecosystem, social justice and equity are the basic requirements of human existence. Community physicians have to advocate consistently for social action to fulfill these prerequisites and mediate between different interests of society so as to enable people to achieve their full health potential. In this respect, the United Nations' Millennium Development Goals provide a comprehensive framework for action.(5)

The effects of ‘market’-oriented development policies on peoples' health need to be investigated in local contexts so as to provide evidence for advocating a reversal of those public policies that do not promote health. Academic programs of Community Medicine should build advocacy skills among health professionals for promoting evidence-based public policies and for mobilizing public opinion in favor of these policies. Building partnerships with civil society organizations and social movements(6) is vital at this stage.

Technological advance in communication, which has led to the shrinkage of space and time, should be harnessed for advocacy, social mobilization and for building alliances at community, national and international levels. Educational opportunities must be enhanced for the creation of not only a technically competent but also socially responsive health workforce. Sufficient number of positions ought to be created for them in the public health system so that all ‘development’ policies are reviewed for their impact on population health and the State can perform its ‘stewardship’ role more effectively in order to achieve the goal of right to health and healthcare for all in the foreseeable future.

The Health Survey and Development Committee, popularly known as the Bhore Committee (after its chairperson Joseph Bhore), had recommended a plan for creation of national health service.(7) Recently, the National Commission on Macroeconomics and Health has also provided a blueprint for the provision of comprehensive primary and secondary healthcare to the Indian population.(8) However, the challenge is to allocate sufficient resources. India consumes about 4.6% of its gross domestic product (GDP) for healthcare but most of the expenditure is privately done by the citizens themselves as the government spends only 0.9% of the GDP on health.(9) Compared to even several developing countries, this is very low and has been declining, but a reversal has occurred recently. The Government of India has planned to raise health-spending to 2–3% to finance the National Rural Health Mission,(10) which is a step in the right direction. But innovation and prudence is required to expand primary healthcare delivery options so that universal coverage is achieved quickly not only in rural areas but also in urban slum populations. An independent monitoring and evaluation system is also required to measure the coverage and impact of the health and development programs.
